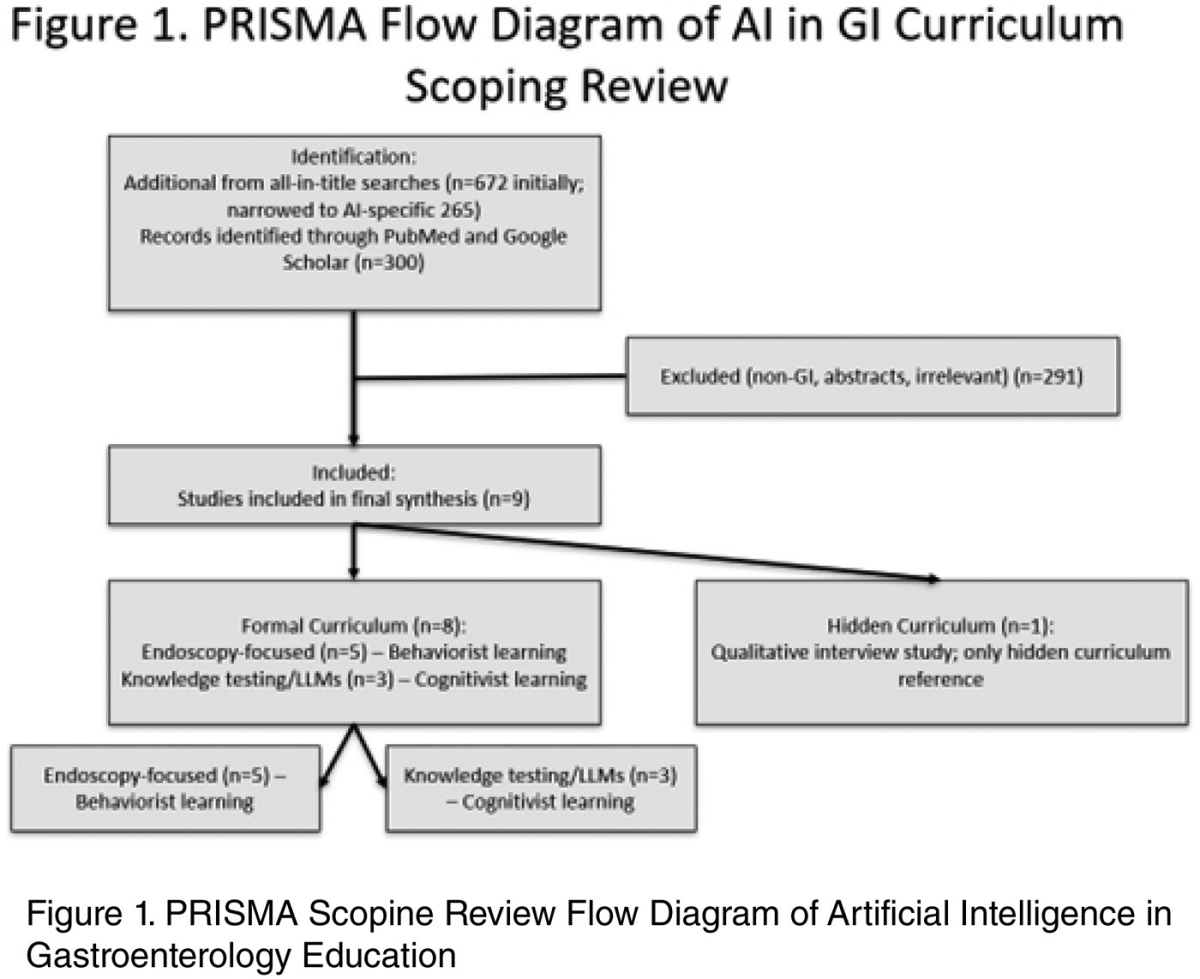# Poster Session I - A132 IMPLICATIONS OF ARTIFICIAL INTELLIGENCE FOR THE FORMAL AND HIDDEN CURRICULUM IN GASTROENTEROLOGY TRAINING

**DOI:** 10.1093/jcag/gwaf042.132

**Published:** 2026-02-13

**Authors:** C Galts, S Samnani, A Wen

**Affiliations:** Gastroenterology, McMaster University, Hamilton, ON, Canada; Gastroenterology, McMaster University, Hamilton, ON, Canada; Gastroenterology, McMaster University, Hamilton, ON, Canada

## Abstract

**Background:**

AI research in GI has recently accelerated, though fewer than 1% of studies involve direct patient contact, raising concerns about ‘dehumanization’ in GI research and training. Given its widespread availability and its influence on learning styles, AI may affect both the formal and hidden curricula.

**Aims:**

We aimed to explore how various AI technologies including Large Language Models (LLMs) and deep learning algorithms, are described in GI training literature, and to identify their implications for both the formal and hidden curricula as well as for relevant learning theories.

**Methods:**

We performed a scoping review using PRISMA-ScR guidelines. PubMed, Embase, and Google Scholar were searched for English-language articles published between January 2010 and June 2025. Search terms included AI-related and GI education terms (e.g., ‘ChatGPT,’ ‘fellowship,’ ‘curriculum’). We used Covidence for title/abstract and full text screening and duplicate removal. Three reviewers filtered the studies, categorized the relevant curriculum (hidden or formal), the type of learning (diagnostic/identification, question based, patient interactions, technical skills) and the associated learning theory since learning theories are rarely explicit in this type of research.

**Results:**

We identified 300 records, and after removal of duplicates and screening, 9 studies met inclusion criteria. Eight addressed the formal curriculum, three of which were reviews, and one referenced the hidden curriculum, which was the only study identified using our search terms that addressed this concept directly. Five studies focused on endoscopic training and reflected primarily behaviorist learning approaches centered on skill acquisition and feedback loops. Three studies examined question banks or knowledge testing, which we classified as drawing mainly on a cognitivist approach to information processing. One study examined fellow perspectives on AI and demonstrated high interest but low uptake of AI tools. Among the review articles, one emphasized cognitive overload and included an example of constructivist learning alongside its behaviorist focus.

**Conclusions:**

Current evidence on AI in GI training centers on endoscopic skill development (behaviorist learning) and LLM-based knowledge acquisition (cognitivist/constructivist learning). There is a gap in research related to the impact of AI on GI training regarding the hidden curriculum. This is despite the fact there is established concern about AI in GI education including issues of misinformation, overreliance, and professionalism. Future studies should examine how AI influences implicit learning and trainee identity formation and compare curricular approaches to optimize benefits while mitigating risks.

**Funding Agencies:**

None